# A Triple Staining Method for Accurate Cell Cycle Analysis Using Multiparameter Flow Cytometry

**DOI:** 10.3390/molecules181215412

**Published:** 2013-12-11

**Authors:** Lin Qiu, Ming Liu, Konglun Pan

**Affiliations:** 1 Institute for Nutritional Sciences, Shanghai Institute for Biological Sciences, Chinese Academy of Sciences, Shanghai 200031, China; E-Mail: qiulin@sibs.ac.cn; 2 Key Laboratory of Marine Drugs, Ministry of Education, School of Medicine and Pharmacy, Ocean University of China, Qingdao 266003, China

**Keywords:** cell cycle, pyronin Y, Hoechst 33342, MPM-2, flow cytometry, crizotinb, taxol

## Abstract

Cell cycle analysis is important for cancer research. We present herein a novel method for accurate cell cycle analysis. This method analyzes the cell cycle by multiparameter flow cytometry based on simultaneously labeling the cell nuclear DNA, RNA, and phosphorylated mitotic nuclei protein, using Hoechst 33342, pyronin Y, and MPM-2-Cy5, respectively, and our results demonstrated that this method could effectively divide the cell cycle into G0, G1, S, G2, and M phases. We further tested this method using the clinical anticancer agents crizotinib and taxol, and the results clearly illustrated that crizotinib and taxol arrested Jurkat cells in G0 and M phase, respectively. These results indicate that this method could be a very useful tool for cytokinetic and pharmacological research.

## 1. Introduction

The cell cycle consists of five distinct phases: G0 phase (quiescence), G1 phase, S phase (synthesis), G2 phase (interphase), and M phase (mitosis). Studies have shown that cancer is a cell cycle-related disease cause by the loss of cell cycle regulation [1,2]. Therefore, cell cycle analysis plays an increasingly important role in the studies of cancer, anticancer agent screening, mechanism illustration, and cytokinetic research [1]. At present, cell cycle analysis by flow cytometry is mainly based on measurements of the DNA content. The traditional single parameter DNA content histogram analysis can only identify G0/G1 phase, S phase, and G2/M phase, but is not able to distinguish G0 from G1 phase or G2 from M phase. For example, crizotinib, a first-in-class dual ALK and c-Met inhibitor, has been shown to be particularly effective against ALK positive non-small-cell lung carcinoma. Using propidium iodide (PI) to measure the DNA content, the single parameter flow cytometry analysis showed that crizotinib arrests non-small cell lung cancer cells in G0/G1 phase [3,4]. The clinically used microtubule inhibitor taxol, arrests A549 cell cycle arrest in the G2/M phases [5]. However, the exact cell cycle arrest phase of these drugs remains unknown, due to the limitations of the present analysis methods. In order to better understand the cell cycle phase arrest, new methods that can accurately divide cell cycle into separate phases are needed.

Several multiparameter flow cytometry analysis methods have been developed based on different expression of cyclins [6,7,8]. Other pioneering studies of cell cycle separation are based on the differences in cellular DNA and RNA content staining with acridine orange/Hoechst 33342 [9] and other dyes. Hoechst 33342 binds DNA without any interaction with RNA, and is widely used in DNA content analysis [10]. Pyronin Y is a stain specific to RNA. In G1 phase, the expression of RNA starts more than in G0 phase, while the DNA still holds diploid. Shapiro *et al*. have developed a method based on pyronin Y/Hoechst 33342 double staining to separate the G0 from the G1 phase [10]. During cell mitosis, many proteins are directly or indirectly phosphorylated by the M phase promoting factor; mitotic phosphoprotein monoclonal antibody-2 (MPM-2) with fluorescent probe conjugate (Cy5) could bind to these phosphorylated amino acid epitopes (such as LTPLK and FTPLQ polypeptide regions), and MPM-2 has been used as a mitosis-specific antibody to distinguish G2 from M cells [11]. Therefore, Hoechst 33342, pyronin Y, and MPM-2-Cy5 have all received much attention in the flow cytometry analysis of the cell cycle. However, most of the flow cytometry analysis using these probes are not multiparameter and there are only a few studies using multiparameter flow cytometry analysis to study the cell cycle [6], and there is no report applying these three fluorescent probe together in cell cycle analysis. According to the characteristics of the three fluorescent probes mentioned above, it is possible that simultaneous application of Hoechst 33342, pyronin Y, and MPM-2 could be established as a novel multiparameter flow cytometry analysis method capable of detecting the five different cell cycle phases.

In the present studies, on the base of Shapiro’s method [10], which stained DNA/RNA with Hoechst 33342/pyronin Y, we employed the third fluorescent probe, MPM-2-Cy5, and established a multiparameter method by simultaneously labeling the cells with Hoechst 33342, pyronin Y, and MPM-2 in flow cytometry analysis and demonstrated the capability of accurately dividing the cells into five groups corresponding to the G0, G1, S, G2, and M phases. We further applied this method to explore the exact cell cycle arrest phase of crizotinib and taxol, and found that they caused Jurkat cells arrest at the G0 and M phase, respectively.

## 2. Results and Discussion

### 2.1. Cells Cycle Analysis by Measuring the DNA Content

**Figure 1:**
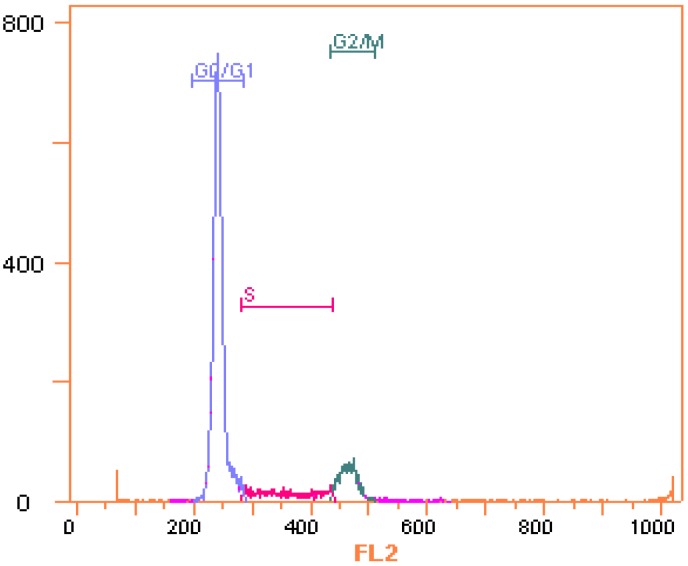
DNA content histogram of Jurkat cell line using single parameter flowcytometry method (PI staining). Cells were ﬁxed in ice-cold 70% (v/v) ethanol for 24 h, collected by centrifugation and stained by a mixture of RNase and PI. The DNA contents of the cells were determined with the Quanta SC flow cytometer (Beckman Coulter, Miami, FL, USA). Data were obtained in three independent experiments and the representative results were shown.

By measuring the DNA content of Jurkat cells using PI staining, the cells cycle distribution was analyzed. As shown in Figure 1, the DNA content histogram showed the cells could be categorized into three groups, G0/G1, S, and G2/M phase. This method entails no cell synchronization and the cell cycle analysis based on DNA content is simple. However, the cell cycle analysis results are inaccurate, because it only considers three groups, G0/G1, S, and G2/M, but cannot distinguish G0 from G1 phase, and G2 from M phase.

### 2.2. Multiparameter Cells Cycle Analysis Divides Jurkat Cell Line in Five Phases

**Figure 2:**
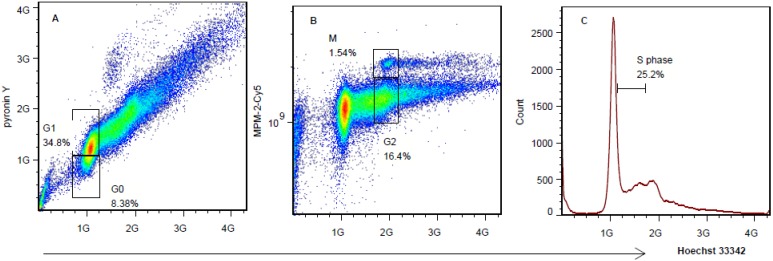
Distribution of cell cycles in Jurkat cells and the bivariate distribution of DNA content (Hoechst 33342), RNA content (pyronin Y), and mitotic phosphoproteins (MPM-2-Cy5 fluoresence) in Jurkat cells. (**A**) The bivariate dot plots showing DNA content (x axis = Hoechst 33342 fluorescence) and RNA content (y axis = pyronin Y fluorescence). The gates were drawn according to the negative control. (**B**) The bivariate dot plots showing DNA content (x axis = Hoechst 33342 fluorescence) and mitotic phosphoproteins content (y axis = MPM-2-Cy5 fluorescence). (**C**) DNA content histogram of Jurkat cell line, stained by Hoechst 33342. Data were obtained in three independent experiments and the representative results were shown. The analysis was carried out without using width/height to delete the doublets.

To overcome the limitations of the DNA content-based cell cycle analysis, we set up a multiparameter flow cytometry analysis using Hoechst 33342, pyronin Y, and MPM-2. As shown in Figure 2, after fixation and permeability, the cells could be divided into five phases, G0, G1, S, G2, and M phase. In Figure 2A, cells in G1 phase (34.8%) could be distinguished from G0 phase (8.38%), because the expression of RNA starts more in G1 phase, and more RNA was stained by pyronin Y, giving a stronger fluorescence in G1 phase cells. Mitotic phosphoprotein is a marker of the mitosis period, and it has been detected by mitosis-specific antibody (MPM-2-Cy5) to distinguish G2 from M cells. In Figure 2B, MPM-2-Cy5 channel, via detection in the fluorescent intensity of MPM-2-Cy5, the cells in G2/M (17.9%) group could be further separated as G2 (16.4%) and M (1.54%) phase, according to the negative control, in which the M phase cells should be Hoechst 33342 (4C) and Cy5 positive cluster cells. The S phase cells (25.2%) were easily separated from the whole cells by simple Hoechst 33342 DNA content analysis, as its DNA content is between 2C and 4C (Figure 2C). This example suggests that the cell cycle of Jurkat cells could be divided accurately into five phases by this multiparameter flow cytometry method we established. The paper published data about Jurkat T cell cycle percentage is that G0/G1 = 52.3%, S = 34.6%, G2/M = 13.2% [12]. Our results are G0 = 8.4%, G1 = 34.8%, S = 25.2%, G2 = 16.4%, M = 1.54%, G0/G1 = 43.2%, G2/M = 17.9%, S = 25.2%, which are similar to the Frank’s [12]. Because the different experimental conditions used, sometimes the results may varies.

### 2.3. Multiparameter Cells Cycle Analysis Divides SMMC-7721 Cell Line in Five Phases

**Figure 3:**
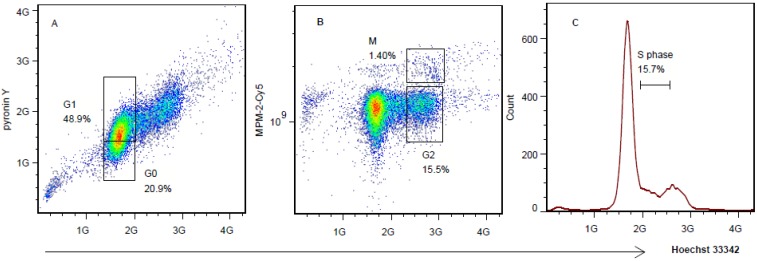
Distribution of cell cycles in SMMC-7721 cells and the bivariate distribution of DNA content (Hoechst 33342), RNA content (pyronin Y), and mitotic phosphoproteins (MPM-2-Cy5 fluoresence) in SMMC-7721 cells. (**A**) The bivariate dot plots showing DNA content (x axis = Hoechst 33342 fluorescence) and RNA content (y axis = pyronin Y fluorescence). The gates were drawn according to the negative control. (**B**) The bivariate dot plots showing DNA content (x axis = Hoechst 33342 fluorescence) and mitotic phosphoproteins content (y axis = MPM-2-Cy5 fluorescence). (**C**) DNA content histogram of SMMC-7721 cell line, stained by Hoechst 33342. Data were obtained in three independent experiments and the representative results were shown. The analysis was carried out without using width/height to delete the doublets.

To further validate this method in other cell lines, an adherent cell line SMMC-7721 was employed. In Figure 3A, the Hoechst 33342/pyronin Y dot plots showed that G1 phase cells gave stronger pyronin Y fluorescence signals. As expected, cells in the G0/G1 (69.8%) group could be separated as G0 (20.9%) and G1 (48.9%) phase. In the Hoechst 33342/MPM-2-Cy5 dot plots (Figure 3B), the cells in the G2/M (16.9%) group also could be further separated as G2 (15.5%) and M (1.40%) phase. The S phase cells were easily separated from the whole cells by simple Hoechst 33342 DNA content analysis (Figure 3C). This method could divide cells into five phases: G0 (20.9%), G1 (48.9%), G2 (15.5%), M (1.40%), and S (15.7%). These results demonstrated that this multiparameter flow cytometry method could be used in both suspending and adhesion cells.

This multiparameter flow cytometry method is based on the increased RNA level in G1 phase and the mitotic phosphoproteins level in M phase. The staining of DNA/RNA by Hoechst 33342/pyronin Y is mainly the same as Shapiro’s method [10]. However, the experimental procedure varies, such as the cellular fixation method and the lasers used to excite the probe, which could avoid adjusting fluorescence compensation and decrease the interference among dyes. Compared with Shapiro’s method, the principal advance in the newly established methods is that, on the base of Shapiro’s method, we employed the third stain, MPM-2-Cy5, and developed a triple staining method and applied it to the accurate cell cycle analysis. This method is also different from previous triple parameter flow cytometric cell cycle analyses, which are mainly based on the expression of cyclins [8,10,11,12]. However, because cyclins expressed unscheduled in some tumor cells [13], those methods are limited to certain cell lines. In addition, the cyclin E+A/DNA method could not distinguish G2 from M phase cells [8]. Although these methods achieved precise separation of G1, S, G2, and M phases, the G0 phase information could not be analyzed. Moreover, the methods based on Cyclin B1/p105/DNA need formaldehyde fixation and could not applied to the cells by alcohol fixation [14]. The multiparameter flow cytometry method we established here is based on the increased RNA level in the G1 phase and the mitotic phosphoproteins level in the M phase. MPM-2 offers the advantage that it works well with alcohol fixation, and therefore could overcome the limitations mentioned above [14]. In our present method, on the previous bases, we employed the MPM-2-Cy5 together with pyroninY/Hoechst 33342, and established a simultaneous triple staining of RNA/MPM-2/DNA, which has not been reported previously. The method could distinguish the inaccurate G0/G1, G2/M phases into precise G0, G1 and G2, M phase in one step on the base of RNA, DNA, and mitotic phosphoproteins levels. However, this method can’t be applied to living cells, thus cell fixation will still be needed. Studies searching for new mitotic protein markers or a new fluorescent probe which could pass across cell membranes to capture mitotic proteins without fixation are in progress.

### 2.4. Crizotinib and Taxol Arrest Jurkat Cells in G0 and M Phase, Respectively

It has been reported that the cell cycle arrest caused by crizotinib is at the G0/G1 phase [15], and that produced by taxol at the G2/M phase [16]. To accurately illustrate the cell cycle arrest of crizotinib and taxol, the newly established multiparameter flow cytometry analysis methods were applied. 

**Figure 4:**
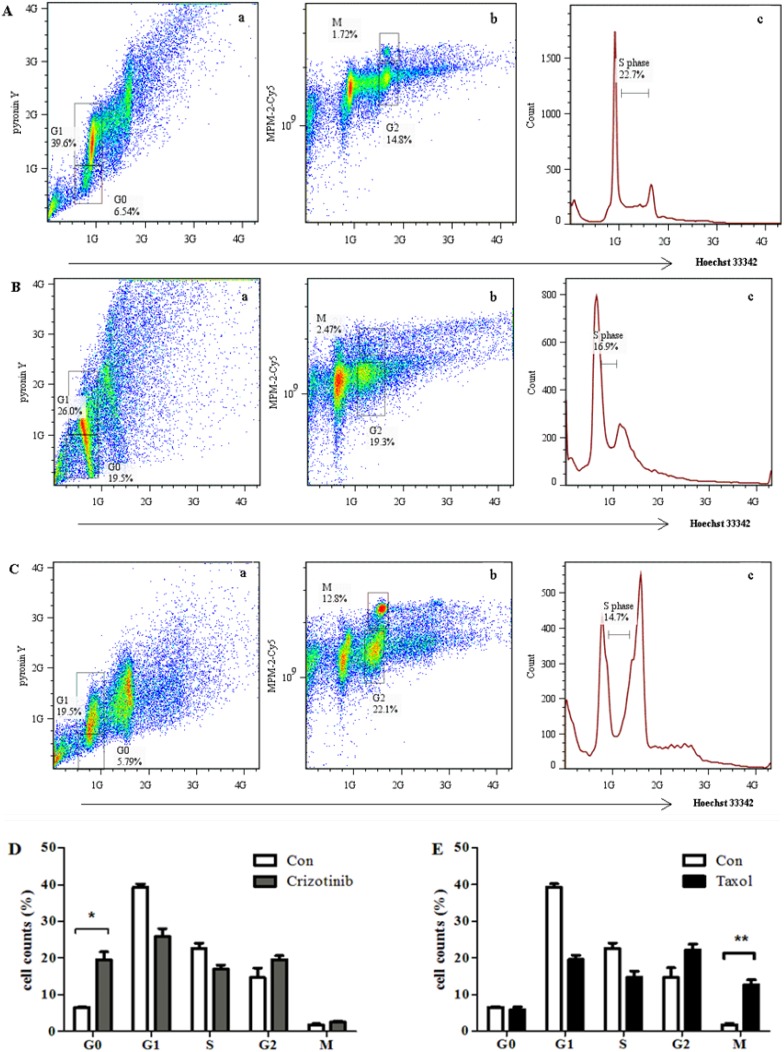
Accurate cell cycle distibution of crizotinib and taxol analyzed by the multiparameter method. (**A**) The bivariate distribution of DNA content (Hoechst 33342), RNA content (pyronin Y) and mitotic phosphoproteins (MPM-2-Cy5) in untreated Jurkat cell. (**a**) The bivariate dot plots showing DNA content and RNA content; (**b**) The bivariate dot plots showing DNA content and mitotic phosphoproteins; (**c**) DNA content histogram of Jurkat cell line, stained by Hoechst 33342. (**B**) The bivariate distribution of DNA content (Hoechst 33342), RNA content (pyronin Y) and mitotic phosphoproteins (MPM-2-Cy5) in crizotinib treated Jurkat cell. (**a**) The bivariate dot plots showing DNA content and RNA content; (**b**) The bivariate dot plots showing DNA content and mitotic phosphoproteins; (**c**) DNA content histogram of Jurkat cell line, stained by Hoechst 33342. (**C**) The bivariate distribution of DNA content (Hoechst 33342), RNA content (pyronin Y) and mitotic phosphoproteins in taxol treated Jurkat cell. (**a**) The bivariate dot plots showing DNA content and RNA content; (**b**) The bivariate dot plots showing DNA content and mitotic phosphoproteins content; (**c**) DNA content histogram of Jurkat cell line, stained by Hoechst 33342. (**D**) Histogram of the cell cycle distribution in the control cells and cells treated with crizotinib. (**E**) Histogram of the cell cycle distribution in the control cells and cells treated with taxol. Values represent the means ± SD. * p values less than 0.05 were considered statistically significant and *p* values less than 0.01 were considered extremely striking. Data were obtained in three independent experiments and the representative results were shown.

As shown in Figure 4A, the untreated Jurkat cells were separated into five G0 (6.54%), G1 (39.6%), S (22.7%), G2 (14.8%), and M (1.72%) groups. When treated with crizotinib, the Jurkat cells in G0 phase increased significantly up to about 20% (Figure 4B), indicating that crizotinib induces cell cycle arrest in Jurkat cells at the G0 phase. When treated with taxol, the Jurkat cells in the M phase cells increased significantly from 1.7% to 12.8% (Figure 4C), indicating that taxol induces cell cycle arrest in Jurkat cells at the M phase. The cell distribution at the five phases with and without the drug treatments are shown in Figure 4D,E. These results suggest that the ambiguous results obtained previously regarding the cell cycle arrest induced by these two drugs, as well as by other anticancer agents, could be further resolved accurately into five phases using this multiparameter method. In addition to the anticancer chemicals studies, the method could also be useful in the cytokinetics area and has potential application in clinical carcinoma diagnosis and prognosis. The major contribution of the present work is to provide a novel triple staining method to discriminate the cell cycle accurately in one step using flow cytometry technology.

## 3. Experimental

### 3.1. Materials and Reagents

Hoechst 33342 and pyronin Y were from Sigma (St.Louis, MO, USA); MPM-2-Cy5 was purchased from Millipore (Billerica, MA, USA); criziotinib (pf-02341066) and taxol were purchased from Selleck Chemicals (Houston, TX, USA). Compounds were dissolved in dimethyl sulfoxide to a final concentration of 10 mM and stored at −20 °C until use.

### 3.2. Cell Lines and Cell Culture

Human T lymphocyte Jurkat cells and human liver cancer cells SMMC-7721 were purchased from Culture Collection of the Chinese Academy of Sciences (Shanghai, China). Jurkat cells are maintained in RPMI 1640 medium supplemented with 10% fetal calf serum (FCS, Gibco, GrandIsland, NY, USA), 100 IU/mL penicillin, and 100 µg/mL streptomycin at 37 °C in a humidified incubator with 5% CO_2_. SMMC-7721 cells are maintained in Dulbecco’s modified Eagle medium (DMEM, Gibco) and the other incubation conditions are the same as for Jurkat cells.

### 3.3. PI Staining and Cell Cycle Analysis

The Jurkat cells were harvested and ﬁxed in ice-cold 70% (v/v) ethanol for 24 h at 4 °C. The cell pellet was collected by centrifugation at 500 × g, resuspended in PBS, and stained with a mixture of RNase (10 µg/mL) and PI (50 µg/mL) in sodium citrate containing 0.5% Triton X-100 for 20 min in the dark. Cell cycle analysis was performed using a Quanta SC flow cytometer (Beckman Coulter, Miami, FL, USA).

### 3.4. Hoechst 33342/Pyronin Y/MPM-2-Cy5 Triple Staining and Cell Cycle Distribution Assays

Cells were fixed in suspension in 0.5% formaldehyde for 15 min at 37 °C in the humidified incubator with 5% CO_2_. The cell suspensions were then centrifuged at 400 g for 5 min and washed once with ice-cold PBS. Then, the cells were vortexed and permeabilized with absolute cold methanol (−20 °C) overnight. After permerabilization, the cells were centrifuged, collected and rinsed once with PBS. The cells were then resuspended in 1 µg/mL Cy5 conjugated MPM-2 antibody in PBS for 1 h at 37 °C. After labeled with the antibody, the cells were rinsed and resuspended in HBSS containing Ca^2+^ and Mg^2+^, at a cell density about 2 × 10^6^/mL. This suspension was finally incubated with equal volume of Hoechst 33342 (2 µg/mL) and of pyronin Y (4 µg/mL) diluted in a solution of Hanks’ balanced salt solution (HBSS) for 20 min.

Cellular fluorescence was measured using a Moflo XDP instrument (Beckman Coulter, Miami, FL, USA) using the following setup parameters: Hoechst 33342 was excited by 355 nm laser, emitted at 450 nm, in FL11 channel, and the signal mode was linar amplifer; pyronin Y was excited by 488 nm laser, emitted at 560 nm, in FL2 channel, and the signal mode was linar amplifer; MPM-2-Cy5 was excited by 633 nm laser, emitted at 650 nm, in FL6 channel, and the signal mode was logarithmic amplifer. The fcs mode data files were analyzed using FlowJo software (Version 7.6.5, TreeStar, Ashland, OR, USA).

The negative control for Jurkat T and SMMC-7721 cells are stained by Hoechst 33342 and IgG-Cy5. We used the bivariate dot plots showing Hoechst 33342/pyronin Y negative control. As there was no pyronin Y in negative control, so all the cells in pyronin Y were negative, and this cluster cell (Hoechst 33342(2C) pyronin Y −) was G0, and the region of the (Hoechst 33342(2C) pyronin Y +) was G1 gate. In addition, we used the bivariate dot plots showing Hoechst 33342/MPM-2-Cy5 negative control. As there was no MPM-2-Cy5 in negative control, so (Hoechst 33342(4C) Cy5–) cluster was set as G2, and (Hoechst 33342(4C) Cy5+) was set as M gate. The cluster in (Hoechst 33342(4C) Cy5+) was the nonsepecific combined cells. At last, S phase was gated (Hoechst 33342(2C–4C)) from the Hoechst 33342 histogram manually, according to the DNA distribution. 

### 3.5. Drug Treatment

Jurkat cells were seeded in 24-well plates. Cells in exponentially growing phase were treated with crizotinib (100 nM) or taxol (25 nM) for 24 h. After the treatment with crizotinib or taxol, the cells were collected by centrifugation at 500 × g, washed and resuspended in PBS. Then the cells were fixed, stained with MPM-2-Cy5, Hoechst 33342, pyronin Y and analysis with flow cytometry as we described above.

### 3.6. Statistical Analysis

Data was expressed as mean ± standard deviation of the mean (SD). Statistics was analyzed using Student’s *t*-test. *p* values less than 0.05 were considered statistically significant and *p* values less than 0.01 were considered extremely striking.

## 4. Conclusions

We have established a novel and alternate multiparameter flow cytometry method for cell cycle analysis by simultaneously labeling the cells with Hoechst 33342, pyronin Y, and MPM-2-Cy5. This method could accurately divide the cells into five groups, corresponding to the G0, G1, S, G2, and M phases, and resolve the ambiguous cell cycle arrest obtained by the single parameter method. Using this newly established method, we showed that the anticancer agent crizotinib and taxol arrested the Jurkat cells at the G0 and M phase, respectively. Our results indicate that this new method could be used for accurate cell cycle analysis in cytokinetic and pharmacological research.
